# Gender discrimination among women healthcare workers during the COVID-19 pandemic: Findings from a mixed methods study

**DOI:** 10.1371/journal.pone.0281367

**Published:** 2023-02-06

**Authors:** Rachel Hennein, Hannah Gorman, Victoria Chung, Sarah R. Lowe

**Affiliations:** 1 Department of Epidemiology of Microbial Diseases, Yale School of Public Health, New Haven, Connecticut, United States of America; 2 Yale School of Medicine, New Haven, Connecticut, United States of America; 3 Department of Social and Behavioral Sciences, Yale School of Public Health, New Haven, Connecticut, United States of America; 4 Yale College, New Haven, Connecticut, United States of America; Oxford University Hospitals NHS Foundation Trust, UNITED KINGDOM

## Abstract

**Background:**

Gender discrimination among women healthcare workers (HCWs) negatively impacts job satisfaction, mental health, and career development; however, few studies have explored how experiences of gender discrimination change during times of health system strain. Thus, we conducted a survey study to characterize gender discrimination during a time of significant health system strain, i.e., the COVID-19 pandemic.

**Methods:**

We used a convenience sampling approach by inviting department chairs of academic medical centers in the United States to forward our online survey to their staff in January 2021. The survey included one item assessing frequency of gender discrimination, and an open-ended question asking respondents to detail experiences of discrimination. The survey also included questions about social and work stressors, such as needing additional childcare support. We used ordinal logistic regression models to identify predictors of gender discrimination, and grounded theory to characterize themes that emerged from open-ended responses.

**Results:**

Among our sample of 716 women (mean age = 37.63 years, SD = 10.97), 521 (72.80%) were White, 102 (14.20%) Asian, 69 (9.60%) Black, 53 (7.4%) Latina, and 11 (1.50%) identified as another race. In an adjusted model that included demographic characteristics and social and work stressors as covariates, significant predictors of higher gender discrimination included younger age (OR = 0.98, 95%CI = 0.96, 0.99); greater support needs (OR = 1.26, 95%CI = 1.09,1.47); lower team cohesion (OR = 0.94, 95%CI = 0.91, 0.97); greater racial discrimination (OR = 1.07, 95%CI = 1.05,1.09); identifying as a physician (OR = 6.59, 95%CI = 3.95, 11.01), physician-in-training (i.e., residents and fellows; OR = 3.85, 95%CI = 2.27,6.52), or non-clinical worker (e.g., administrative assistants; OR = 3.08, 95%CI = 1.60,5.90), compared with nurses; and reporting the need for a lot more childcare support (OR = 1.84, 95%CI = 1.15, 2.97), compared with reporting no childcare support need. In their open-ended responses, women HCWs described seven themes: 1) belittlement by colleagues, 2) gendered workload distributions, 3) unequal opportunities for professional advancement, 4) expectations for communication, 5) objectification, 6) expectations of motherhood, and 7) mistreatment by patients.

**Conclusions:**

Our study underscores the severity of gender discrimination among women HCWs. Hospital systems should prioritize gender equity programs that improve workplace climate during and outside of times of health system strain.

## Introduction

Although women comprise the majority of the healthcare workforce, holding 76% of all healthcare positions [1], gender discrimination against women healthcare workers (HCWs) is widespread. Previous studies have estimated that 66–80% of women HCWs have experienced gender discrimination in the workplace [2, 3]. Gender discrimination in the hospital can take the form of sexual harassment [3], inequitable compensation [4–8], diminished career advancement to leadership roles [9, 10], and the misidentification of women physicians’ roles [3]. Gender discrimination can be perpetuated by individuals of all genders, and by the health system itself (such as through gendered policies). Gender discrimination negatively impacts women HCWs’ interactions with patients [11], job satisfaction [10], and mental health [12].

Women HCWs, compared with men HCWs, are at increased risk for psychological distress during the COVID-19 pandemic [11–13], with one potential explanation being their heightened exposure to gender-based stressors. For example, a survey study including frontline HCWs at an academic hospital in New York City during the COVID-19 pandemic found that women HCWs were more likely than men HCWs to screen positive for depression, anxiety, and PTSD [13]. This increased risk of adverse mental health outcomes was largely attributed to background stressors for women that were exacerbated during the pandemic, including burnout and family-related concerns [13]. Identifying other background stressors, such as gender discrimination, is important to comprehensively understand why women HCWs have been particularly impacted by the pandemic.

The COVID-19 pandemic has acted like a “stress test” for hospitals, exposing inequitable and unsustainable structures and practices within the health system [14]. Studying experiences of gender discrimination during the pandemic can assist in identifying important areas for improvement during and outside of the current health crisis. For example, the pandemic has highlighted how the dual responsibilities of being a HCW and mother are strained when hospital surges are coupled with childcare shortages, providing an opportunity to advocate for improved access to childcare even after the pandemic subsides [15].

Conceptualizing the pandemic as a “stress test,” we conducted a mixed methods survey study of women HCWs to characterize gender discrimination during a time of significant health system strain. In so doing, we aimed to identify the magnitude of and factors associated with gender discrimination in the hospital, as well as narratives of these experiences to inform opportunities for change.

## Materials and methods

### Setting and recruitment

This study is part of a larger project that assesses the psychological impact of the COVID-19 pandemic on HCWs. We purposively sampled from 25 academic hospitals located in 14 states with the highest weekly incidence rates of COVID-19 based on real-time data [16]. By doing so, we included hospitals that were particularly strained due to high burden of COVID-19 in their catchment area. These hospitals are distributed across all regions of the US, including the Northeast, South, Midwest, and West. We used a serial cross-sectional design, whereby we collected data every six months beginning in June 2020 from different samples of HCWs in the United States (US) [17]. The present study is a secondary analysis that focuses on the items related to gender discrimination, and uses primary data that were collected from December 1, 2020 until January 14, 2021 when we met our recruitment target of 900 HCWs based on power calculations. During this period, there were 139,152 to 314,093 new cases of COVID-19 per day in the US [18].

We used a convenience sampling approach by emailing department chairs, such as the Chair of Internal Medicine, to request that they forward our web-based survey to their staff. We obtained contact details for these chairs using academic hospitals’ websites. Of the 298 department chairs we invited, 122 agreed to participate, four declined to participate, and 172 did not respond. The reason that department chairs declined to participate was survey fatigue, as their staff were asked to complete numerous surveys on their wellbeing at the time. We employed two inclusion criteria: 1) participants were at least 18 years of age; and 2) participants worked at a clinic/hospital, including physicians, physicians-in-training (i.e., residents and fellows), nurses, health technicians, physician assistants, nursing/medical assistants, other clinical workers, and non-clinical workers. Although the survey collected responses from men (n = 261) and a small subsample of participants identifying as a gender minority (n = 9), the present study only includes women HCWs.

### Data collection

We used a convergent parallel mixed methods design by collecting and analyzing quantitative and qualitative data simultaneously, then merging qualitative and quantitative results [19]. We designed a web-based survey to include quantitative and qualitative questions on gender discrimination and hypothesized risk and protective factors (S1 File). All data were collected using the Qualtrics platform.

#### Gender discrimination

We assessed gender discrimination using a single item that assessed how often respondents have been treated unfairly based on their gender in the previous year, since January 2020. Response options ranged from never (score = 1) to almost all the time (score = 6). This item has been previously used to assess gender discrimination among women, men, and gender minorities [20]. Respondents who reported any frequency of gender discrimination (i.e., score>1) were presented with an additional open-ended question asking them to describe an event in which they dealt with gender discrimination.

#### Covariates

We selected covariates based on previous studies that identified factors associated with gender discrimination among HCWs [21–24], as well as demographic characteristics (i.e., age, race, ethnicity, marital status, and occupation). For example, previous studies have found that gender discrimination is associated with childcare needs [23] and racial discrimination [21, 22]. We assessed childcare needs by asking respondents who had children if they needed a lot (score = 3), a little (score = 2), or no more (score = 1) childcare support. The childcare support variable was coded as two indicator variables, with no more additional childcare support used as the reference category. We used the General Ethnic Discrimination Scale to assess experienced racism in the past year [25]. This scale includes 18 items that assess various experiences of racial discrimination. We included an additional item asking about racial discrimination from patients. All items were scored on a 6-point Likert scale, from never to almost all the time, and were summed to create a continuous racial discrimination score (range: 19 to 114). This scale has been validated among Black, Latinx, Asian, and White respondents [25]. We also assessed social factors to understand how gender discrimination relates to perceived social support and team cohesion. Social support was measured using one item from the Social Support Questionnaire from the National Health and Nutrition Examination Survey to assess if the participant needs a lot (score = 4), some (score = 3), a little (score = 2), or no more social support (score = 1) [26]. The social support score was included as a continuous variable. Team cohesion was measured using previously validated questions from the Survey of Organizational Attributes for Primary Care to assess communication, decision making, and stress/chaos [27, 28]. This scale includes seven questions, each rated on a five-point Likert scale from strongly disagree to strongly agree. The scale score is computed by summing all items, and ranges from 7 to 35.

### Data analysis

#### Quantitative analysis

We first conducted a missing data analysis to compare participants included in the analytic sample with those who were dropped due to missing data using independent-samples t-tests and chi-square analyses [29]. We assessed correlations between all independent variables to assess for multi-collinearity and data suitability for analysis. After ensuring the multi-collinearity assumption was not violated, we ran unadjusted ordinal logistic regression models to test bivariate associations between gender discrimination and all factors [29]. Lastly, we ran an adjusted ordinal logistic regression model to predict gender discrimination, which included age, race, ethnicity, marital status, occupation, social support needs, team cohesion, childcare needs, and racial discrimination as covariates [29]. Analyses were conducted in SPSS 27.0 (IBM Corp., 2020). We considered p < .05 to be statistically significant.

#### Qualitative analysis

We uploaded all responses to the open-ended question into Microsoft Excel. Our coding team included a woman doctoral student and a woman Master of Public Health student (RH and HG, respectively). We coded all data using inductive approaches based on grounded theory to develop codes and ultimately theory that describe how women experience gender discrimination in healthcare professions [30]. First, we read all responses to generate preliminary, inductive codes. We then met to develop the codebook by explicating definitions, inclusion criteria, and exclusion criteria for each code. Using this codebook, we independently coded the first 50 open-ended responses and met to discuss discrepancies and make necessary modifications to the codebook. Once the codebook was finalized, we independently coded the entire set of responses. We used Cohen’s Kappa to calculate intercoder reliability [31]. We defined sufficient intercoder reliability as Cohen’s Kappa>0.80. Then, we discussed any discrepancies in our coding to reach consensus and generate the final coded datafile. Finally, we met to discuss relationships between codes to conceptualize broad themes using thematic analysis [26]. We used reflexivity throughout the coding process by reflecting on how our own experiences and identities could influence our interpretations of the data [32].

#### Merging quantitative and qualitative findings

**Fig 1:**
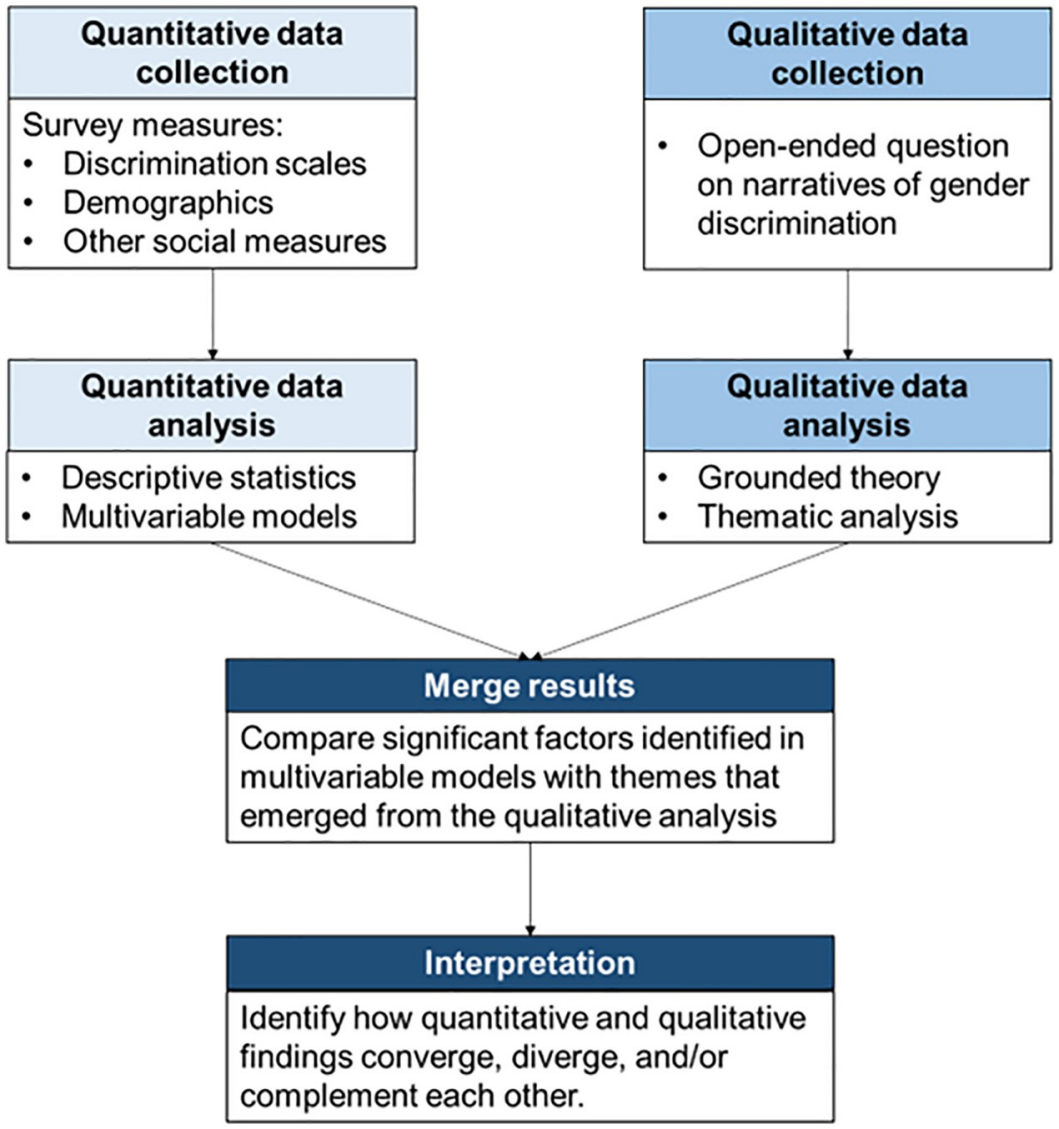
Mixed methods design.

To merge our quantitative and qualitative findings, we mapped themes that emerged from the qualitative analysis to significant factors identified in the adjusted ordinal logistic regression model (Fig 1). We then made interpretations by identifying how quantitative and qualitative findings converged, diverged, and/or complemented each other [19]. We used the Good Reporting of A Mixed Methods Study (GRAMMS) checklist to guide study reporting [33].

#### Ethics

The Yale Institutional Review Board approved our study and all participants provided written consent electronically before taking the survey. The first page of the online survey was a consent form, which the participant had to agree to before being directed to the main survey.

## Results

### Study sample

Among the 752 women who took our survey, 36 (4.8%) had missing data for at least one of the variables. We did not identify any significant differences between participants in the analytic sample (n = 716) and those who were dropped. Among the 716 women who completed all measures, 521 (72.80%) were White, 102 (14.20%) Asian, 69 (9.60%) Black, 53 (7.4%) Latina, and 11 (1.50%) identified as another race, including American Indian/Alaska Native, Native Hawaiian/Pacific Islander, and unspecified (Table 1). The average age of our sample was 37.63 years (*Standard Deviation [SD]*: 10.97). Most women (n = 515; 71.90%) did not have a child who required childcare. Among those with a child who required childcare (n = 201), 45 (22.39%) did not need any additional childcare support, 74 (36.82%) needed a little more childcare support, and 82 (40.80%) needed a lot more childcare support. More than half of women (56.01%) reported experiencing gender discrimination in the past year.

**Table 1:** Participant characteristics.

Characteristic	n (%) or Mean (SD)
Age^a^	37.63 (10.97)
Race^b^	
White	521 (72.8%)
Asian	102 (14.2%)
Black	69 (9.6%)
Other^c^	11 (1.5%)
Ethnicity^b^	
Latinx	53 (7.4%)
Non-Latinx	663 (92.6%)
Marital status^b^	
Married	383 (53.5%)
Single	287 (40.1%)
Divorced/widowed	46 (6.4%)
Occupation^b^	
Physician	185 (25.8%)
Physician-in-training	182 (25.4%)
Nurse	113 (15.8%)
Health technician	66 (9.2%)
Physician, nursing, and medical assistant	42 (5.9%)
Other clinical worker	73 (10.2%)
Non-clinical worker	55 (7.7%)
Social support needs^a^	1.44 (1.05)
Has child that requires childcare^b^	201 (28.1%)
Childcare needs among those with a child (n = 201)^b^	
No additional childcare support needed	45 (22.4%)
A little more childcare support needed	74 (36.8%)
A lot more childcare support needed	82 (40.8%)
Team cohesion^a^	24.51 (4.71)
Gender discrimination^a^	1.85 (0.96)
Racial discrimination^a^	23.73 (8.44)

Abbreviations: SD, standard deviation.

^a^The mean and standard deviation are reported for continuous variables.

^b^The counts and percentages are reported for categorical variables.

^c^Other race included: American Indian/Alaska Native, Native Hawaiian/Pacific Islander, and unspecified

### Quantitative findings

In the unadjusted models, age, race, marital status, occupation, social support needs, team cohesion, childcare needs, and racial discrimination were associated with reporting gender discrimination (Table 2). After adjusting for all factors, younger age (Odds Ratio [OR] = 0.98, 95% Confidence Interval [CI] = 0.96, 0.99), greater social support needs (OR = 1.26, 95%CI = 1.09,1.47), lower team cohesion (OR = 0.94, 95%CI = 0.91, 0.97), and greater racial discrimination (OR = 1.07, 95%CI = 1.05,1.09) were associated with greater gender discrimination. Additionally, Black women reported less gender discrimination than White women after adjusting for all factors including racial discrimination (OR = 0.52, 95%CI = 0.30, 0.92). Compared with nurses, physicians had 6.59 odds (95%CI = 3.95, 11.01), physicians-in-training 3.85 odds (95%CI = 2.27,6.52), and non-clinical workers 3.08 odds (95%CI = 1.60,5.90) of reporting greater gender discrimination. Compared with not needing any additional childcare support, needing a lot more was associated with greater gender discrimination (OR = 1.84, 95%CI = 1.15, 2.97).

**Table 2:** Unadjusted and adjusted models predicting gender discrimination.

Covariate	Unadjusted OR (95%CI)	Adjusted OR (95%CI)
Age	0.97 (0.96,0.98)***	0.98 (0.96, 0.99)**
Race		
White (reference)	1	1
Asian	1.85 (1.25,2.74)**	0.78 (0.50,1.22)
Black	1.36 (0.86,2.17)	0.52 (0.30, 0.92)*
Other^a^	1.46 (0.49,4.36)	1.36 (0.41,4.55)
Ethnicity		
Non-Latinx (reference)	1	1
Latinx	1.66 (0.99,2.78)	0.97 (0.56,1.71)
Marital status		
Married (reference)	1	1
Single	1.37 (1.03,1.82)*	1.31 (0.91,1.90)
Divorced/widowed	0.58 (0.32,1.07)	0.93 (0.48,1.80)
Occupation		
Nurse (reference)	1	1
Physician	5.06 (3.15,8.14)***	6.59 (3.95,11.01)***
Physician-in-training	4.79 (2.97,7.71)***	3.85 (2.27,6.52)***
Health technician	1.20 (0.64,2.25)	1.09 (0.56,2.11)
Physician, nursing, and medical assistant	1.65 (0.81,3.33)	1.42 (0.68,2.98)
Other clinical worker	1.41 (0.77,2.56)	1.41 (0.75,2.64)
Non-clinical worker	3.02 (1.61,5.65)**	3.08 (1.60,5.90)**
Support needs	1.45 (1.27,1.66)***	1.26 (1.09,1.47)**
Team cohesion	0.95 (0.92,0.98)**	0.94 (0.91, 0.97)***
Childcare needs		
None (reference)	1	1
A little	0.97 (0.62,1.53)	0.98 (0.59,1.62)
A lot	2.01 (1.31,3.07)**	1.84 (1.15,2.97)*
Racial discrimination	1.06 (1.05,1.08)***	1.07 (1.05,1.09)***

*p < .05 and > = .01;

**p < .01 and > = .001;

***p < .001

^a^Other race includes: American Indian/Alaska Native, Native Hawaiian/Pacific Islander, and unspecified

Abbreviations: OR, Odds Ratio; 95%CI, 95% Confidence Interval.

### Qualitative findings

Women HCWs described seven major themes related to experiencing discrimination based on their gender in their workplace: 1) belittlement by colleagues, 2) gendered workload distribution, 3) unequal opportunities for professional advancement, 4) expectations for communication, 5) objectification, 6) expectations of motherhood, and 7) mistreatment by patients. Cohen’s Kappa for each code ranged from 0.84 to 1.00, suggesting sufficient intercoder reliability.

#### Belittlement by colleagues

First, many respondents reported feeling belittled when their colleagues made gendered assumptions about their profession and used the incorrect professional title. For example, women physicians reported that they were often assumed to be nurses by their colleagues. Even when colleagues knew of their profession, many would not address women physicians as “doctor,” whereas men physicians were addressed by their proper title. A Black/African American physician-in-training explained:

“*Nurses*, *technicians*, *residents*, *other attending physicians always assume I am a nurse*, *based primarily on gender*, *age*, *and race I would imagine- it happens countless times per day and if I had the time to reflect on it while it happened*, *I wouldn’t have time to do anything else as it happens so often*.”

This respondent, in addition to others, suggested that being misidentified was due to a combination of their gender, race, and age, suggesting that discrimination experiences among HCWs are interpreted with respect to their intersecting identities.

Additionally, many women reported that their men colleagues used condescending language and had a dismissive tone during conversations about work issues. A Black/African American physician explained:

“*As an attending hospitalist*, *I often have interactions with subspecialty attendings*, *particularly when challenging patient care issues arise*. *Frequently*, *those attendings are older white men*. *Many times*, *I’m spoken to in what I perceive as a condescending*, *belittling or dismissive manner during these conversations*, *particularly when I’m advocating a position with which that person disagrees*. *I’ve witnessed my male peers having similar conversations with those same individuals that are far more productive and collegial*. *These interactions make me feel unvalued and like a lesser member of the faculty than my equally-titled male peers*.”

Despite her expertise, this physician described that when she advocates for her patients, her ideas are dismissed. However, when “male peers” have similar conversations, they are treated in a more collegial and professional way. This suggests that women HCWs are not given the same credibility as men, which leads to feelings of being undervalued.

#### Workload distribution

Women reported that their workload distribution, both the content of their work tasks and hours, was gendered. Many respondents reported that they were often given additional roles or responsibilities that they interpreted as gendered, including organizing social events, secretarial work, and cleaning. For example, one physician wrote:

“*I’ve been given more secretarial tasks rather than my male colleagues who were given leadership tasks*. *My work was not visible or celebrated*.”

Although this HCW is a physician and is thus qualified to take on hospital leadership tasks, she is instead delegated secretarial tasks. This differential allocation of work tasks may impact the ability of women HCWs to gain leadership experiences, and ultimately to be promoted to leadership roles in their workplaces.

Women respondents also reported that they were asked to take on additional work compared with men, which was oftentimes unpaid and unacknowledged. A nurse explained:

“*Our APP [Advanced Practice Providers] team was all women*. *We were expected to work OT [overtime] to continue to staff the COVID ICU [intensive care unit] even when much of the hospital had returned to normal operations*. *There was no acknowledgement of the work we had done throughout the pandemic despite other teams getting recognition*. *It felt very gendered that there was the expectation that we’d keep doing unrecognized labor and OT [overtime]*”

This nurse described an implicit assumption that her majority-women team would work overtime throughout the pandemic. Due to this assumption, her team failed to be recognized for the sacrifices that they made throughout the pandemic. Thus, gender-based assumptions about willingness and ability to take on additional work is an important form of gender discrimination.

#### Unequal opportunities for professional advancement

Women reported that they faced unequal opportunities for professional advancement such as salary, promotions, and job opportunities. Many reported being grossly underpaid compared with men, which made them feel undervalued. A Black/African American and American Indian/Alaska Native lab technician explained:

“*I have been passed over for lead positions by those less qualified and with less seniority*. *I believe this occurred because the other person is a young white male and I am a Female POC [person of color]*. *I also make less money than my white male counterpart in the same lab whom also has less experience and seniority than myself*.”

This respondent again exemplifies the importance of considering both gender and race when evaluating forms of discrimination in the workplace, as she perceives that unequitable opportunities for promotion and pay on her team are due to her intersecting identities as a woman and person of color.

Respondents also noted unfair hiring of new personnel based on gender. A health technician explained:

“*Even when the female applicant had more experience and performed more work than the male applicant*, *the male applicant was selected for job promotion*. *It felt unfair*.”

This woman, in addition to others, reported blatant favoritism to advance the careers of men, such as through hiring, promotions, and pay raises.

#### Expectations for communication

Respondents reported conflicting, gendered expectations for how women should behave and speak at work. For example, some women were told to be less timid, while others were assumed to be aggressive:

“*There seems to be a perception that women who speak normally are too timid—why is there a need to be extra loud/abrasive to be heard*? *Confidence and competence come in many forms*.”

*(physician-in-training)*

“*Called aggressive when expressed ideas that if one of my male colleagues would have expressed would have been considered assertive*.”

*(physician)*

The Asian physician-in-training was described as too timid, while the Black/African American physician was called aggressive. In this way, beliefs about how women HCWs should communicate are not only conflicting, but also function to prevent women from communicating in their own style.

Many respondents also reported that they were critiqued for being too “emotional.” A physician shared:

“*I was told that female faculty members of my division were ’emotional’ in their asks for additional staffing support during administrative meetings*. *This made me feel angry that all voices were not being heard*.”

This HCW described that being labeled as “emotional” functioned to silence women HCWs from voicing their opinions on important staffing decisions.

#### Objectification

Women reported experiences of objectification in the workplace. For example, they reported that colleagues made comments that communicate a fixation with their appearance, rather than their performance. A physician shared:

“*People are very fixated on my weight in a way that I think would not happen if I were a man*. *I have lost weight but have a healthy BMI [body mass index] and everyone keeps bringing it up with me*. *It makes me feel that my appearance counts more than my performance*.”

This physician described that frequent comments fixating on her weight signal to her that her appearance is more important than her performance in the hospital.

Many respondents also reported being explicitly sexually harassed in the workplace and not receiving adequate support following these experiences. A lab technician explained:

“*I was sexually harassed by a coworker who made frequent explicit sexual commentary along with demeaning commentary about women (saying he hated women*, *using gendered slurs*, *complaining about female doctors)*. *When I reported this to my boss*, *I was placed alone with the offending party on a COVID adjusted night shift schedule*.”

This lab technician’s account of her experience and supervisor’s response underscores the minimization of reports of sexual harassment in the workplace.

#### Expectations of motherhood

Respondents reported experiencing certain motherhood expectations that colleagues who are men did not face. For example, respondents reported that their colleagues assumed that their work would be negatively impacted by their role as a mother. A psychologist explained:

“*Some senior colleagues questioned whether I would return to work from a planned maternity leave based on the suggestion that women often don’t*. *I felt underappreciated and patronized*.”

Questioning women HCWs’ decisions to come back to work after maternity leave inappropriately asserts gender-based expectations for childrearing. For this respondent, these comments made her feel not only patronized, but also underappreciated.

Many respondents also noted how addressing childcare shortages they faced due to the pandemic and simultaneous added workload in the hospital, which disproportionately impacted women, was not prioritized by hospital administration. A physician explained:

“*Leadership did not understand the dire childcare situation*. *The COVID-19 pandemic will set back accomplishments of women in the workplace for decades to come*.”

Notably, respondents who were not presently mothers also reported certain expectations related to their childbearing. A physician-in-training shared:

“*It was assumed that I wanted to pursue anesthesiology because it ‘allows for more flexibility to raise children*.*’ I currently have no desire to have children*… *though this could be considered an innocent assumption*, *it just happens to be a constant stigma women in medicine must battle against and are hyper aware of*.”

This account shows that HCWs’ experience in the gender discrimination related to motherhood could contribute to distress, including hypervigilance.

#### Mistreatment by patients

Lastly, respondents expressed that they faced mistreatment by patients based on their gender. For example, they reported that their patients often assumed they were a different profession based on their gender; namely, women physicians were assumed to be nurses. A physician described:

“*Often assumed to be a nurse*, *or not the doctor*. *Two times when patient assumed male med [medical] student was the doctor and asked if he ‘approved’ of my recommendations*. *Both situations made me feel frustrated and disappointed*.”

This physician’s recollection demonstrates how gender discrimination from patients can influence women HCWs’ emotional wellbeing, perhaps undermining job satisfaction.

Women noted that even after they told patients that they were the physician, many would still not address them as a doctor, and some would even refuse to be seen by a woman physician. A physician-in-training recalled:

“*I had an instance where a patient requested their ‘real doctor’*. *They were not satisfied until a male colleague intervened*. *As a woman in medicine this is not an unfamiliar encounter*. *It is disheartening but even more so in this pandemic where I am working harder than ever for my patients*.”

This account demonstrates that gender discrimination, although present prior to the pandemic, was particularly hurtful given the profound sacrifices women HCWs were making for their patients.

Notably, many women suggested that they were treated unfairly by patients based on a combination of their gender and ethnicity. A Latina physician-in-training explained:

“*Taking care of a patient at the VA [Veterans Affairs Hospital]*, *he said that women should not be doctors*, *and called me a foreigner*. *He then walked out of the room and refused being seen by me*. *Like that*, *many other similar experiences*.”

This HCW described how patients frequently refuse to be seen by her due to a combination of her gender and ethnicity, underscoring the importance of considering intersecting identities to interpret discrimination experiences. These common occurrences suggest that there is a lack of mechanisms for reporting instances of patient-perpetuated discrimination.

### Merged findings

**Fig 2:**
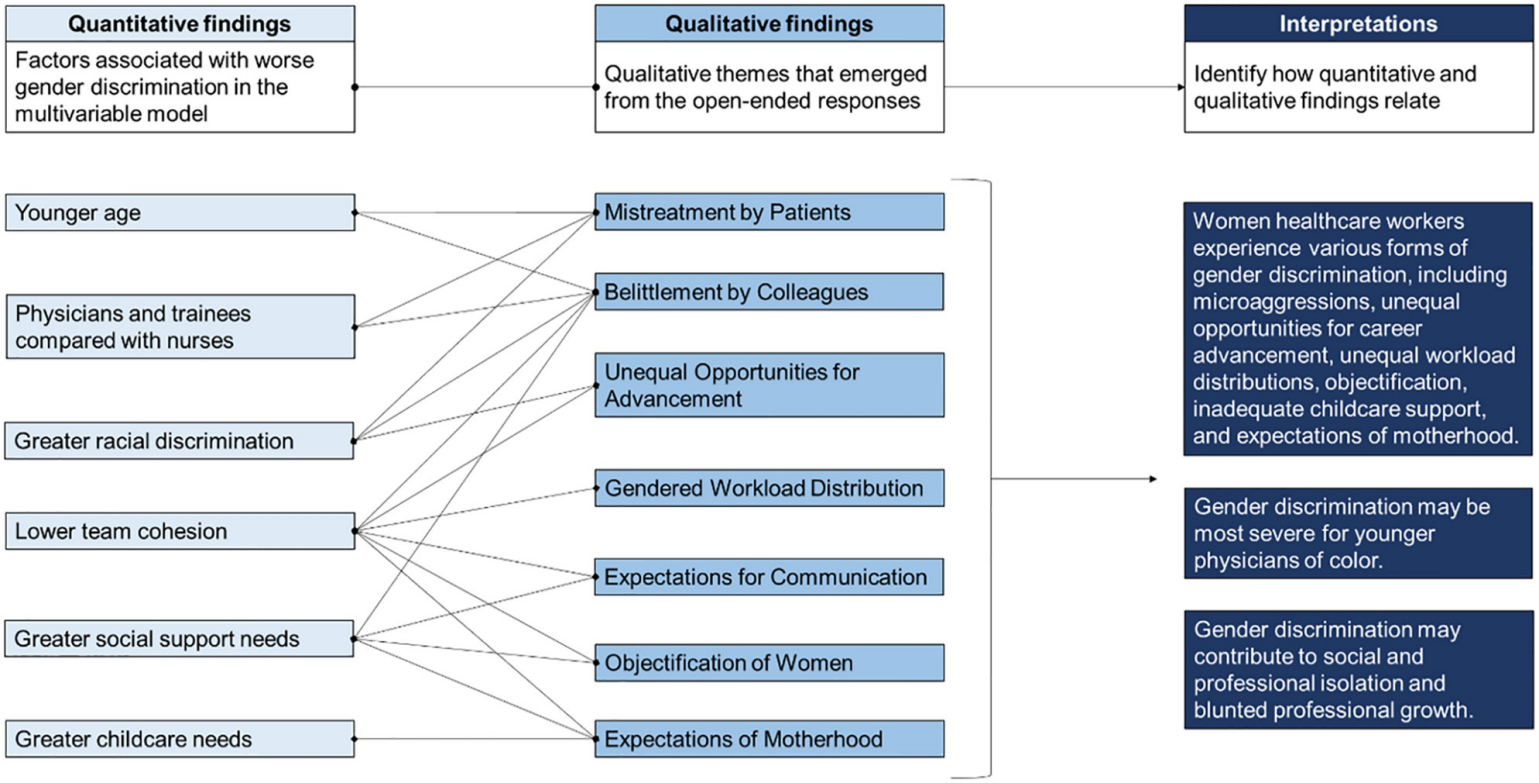
Merging of quantitative and qualitative findings.

We mapped themes that emerged from the qualitative analysis to significant factors identified in the multivariable model (Fig 2). For example, in open-ended responses, many women HCWs described being belittled by their colleagues and supervisors, which made them feel unappreciated by their team. Accordingly, this theme mapped onto the finding from the multivariable model that lower team cohesion was associated with heightened gender discrimination. This mapping process enabled us to identify three ways that the quantitative and qualitative findings merged to characterize gender discrimination among women HCWs.

First, through our qualitative analysis, we found that women HCWs experienced various forms of gender discrimination from both patients and colleagues. These experiences included belittlement, unequal opportunities for career advancement, gendered workload distributions, expectations for communication, objectification, and expectations of motherhood. Some of these experiences were occupation-specific; for example, many women physicians noted that they were frequently misidentified as a nurse or custodial staff by colleagues and patients. Our quantitative and qualitative findings converged regarding motherhood expectations; in open-ended responses, women reported that having inadequate childcare support during the pandemic was a form of gender discrimination and needing a lot more childcare support was associated with higher gender discrimination in the multivariable model. Our qualitative findings further elaborated on the far-reaching implications of motherhood expectations, which extended to women HCWs who were not currently mothers.

Second, we identified that gender discrimination may be most severe for younger physicians of color. Our multivariable model suggested that younger HCWs, HCWs who experienced greater racial discrimination, and HCWs in occupations that are men-dominant (i.e., physicians and physicians-in-training) reported more frequent gender discrimination. Although race was not a significant predictor of gender discrimination in the unadjusted model, Black women compared with White women, were less likely to report gender discrimination after controlling for all factors including racial discrimination. This finding could suggest that experiences of gender discrimination among Black women are also influenced by experiences of racial discrimination. These quantitative findings converged with our qualitative findings; in the open-ended responses, women HCWs reported that their experiences of gender discrimination are likely due to a combination of their age, gender, race, ethnicity, and role as a physician.

Lastly, we identified that gender discrimination was associated with lower team cohesiveness and inclusiveness, which could contribute to social and professional isolation and limitations to professional growth. This interpretation was based on our quantitative findings that greater gender discrimination was associated with decreased team cohesion and increased social support needs. Our qualitative findings elaborated on these quantitative findings by describing how frequent microaggressions, harassment, and inappropriate assumptions about motherhood impacted their relationships with colleagues. Women HCWs also described that unequal career opportunities and workload distributions negatively impacted their professional growth.

## Discussion

This mixed methods study explored gender discrimination among women HCWs. In our quantitative analysis, we identified factors associated with higher reports of gender discrimination, including younger age, certain occupations (i.e., physicians and physicians-in-training, versus nurses), greater racial discrimination, lower team cohesion, greater social support needs, and greater childcare needs. Our qualitative analysis bolstered these findings by describing how gender discrimination is experienced in the hospital and how it impacts women HCWs. By merging our quantitative and qualitative data, we described how gender discrimination can present itself through various forms, may be most severe for younger physicians of color, and how it can contribute to social and professional isolation and blunted professional growth. Our findings expand upon previous studies of gender discrimination among HCWs and can be used to change hospital systems to improve inclusivity of women in healthcare.

Women HCWs suggested that they experience various forms of gender discrimination perpetuated by both patients and colleagues. Similar to previous studies, women HCWs reported incidences of sexual harassment [3]; microaggressions [21, 34]; expectations of motherhood [2, 34]; and unequal career advancement, compensation, and workload distributions [4–6, 9, 23, 34]. We found that certain forms of discrimination may only be applicable to certain professions; for example, women physicians reported being frequently mistaken for nurses, which is consistent with other studies [3, 10, 11].

Whether from patients or colleagues, gender discrimination takes a significant toll on the wellbeing of women HCWs. Our findings suggested that some of these experiences of gender discrimination may be more pronounced during the pandemic, such as shortages in childcare and lack of support from the health system for HCWs who are mothers, similar to other studies [24, 35, 36]. Gender discrimination during the pandemic may be particularly damaging given the profound sacrifices made by women HCWs. For example, in the present study, women HCWs reported that these experiences of gender discrimination made them feel “disheartened,” “unvalued,” “unrecognized,” “disappointed,” “angry,” and “hopeless.” Other studies have found that gender discrimination is associated with adverse mental health outcomes [12, 21], and makes women feel less satisfied with their jobs, less respected by patients and colleagues, and that their gender impacts their opportunities for career advancement [10, 11]. Based on these findings, responses to health emergencies should include women’s perspectives on support services that are needed, such as enhanced childcare services, which may offset stressors disproportionately impacting women during health crises [24, 36, 37].

We identified that gender discrimination may be most severe for younger physicians of color, as age, gender, occupation, and experiences of racism were associated with gender discrimination. Women physicians of color suggested that they are commonly misidentified as nurses or custodial staff by patients and colleagues, which they attribute to a combination of their age, gender, ethnicity, and race. Furthermore, we found that non-clinical HCWs had higher reports of gender discrimination compared with nurses. This trend reflects the importance of intersectionality in understanding women’s experiences of discrimination. The theory of intersectionality describes how multiple social identities, such as gender, race, ethnicity, occupation and age, interact to influence experiences of discrimination. This theory was first proposed by Kimberlé Crenshaw to describe the unique experiences of discrimination that Black women face in comparison to White women or Black men [38]. Other studies have also described the importance of considering multiple social identities to understand experiences of discrimination faced by women HCWs [39, 40]. For example, another study that utilized quantitative and qualitative methods to explore racial discrimination among HCWs identified that experiences of racial and ethnic discrimination were most common among HCWs of color, and that these experiences were interpreted in light of intersecting identities, namely gender, race, and ethnicity [41]. Another study including medical students found that microaggressions were most frequent among Black women, compared with Black men and White women [21]. These findings suggest that different power dynamics for subgroups of women may be at play that influence women’s experiences of gender discrimination. Future studies could explore varying power dynamics across different subgroups and settings, such as how gender discrimination operates based on an individual’s occupation and racial identity, as well as the gender and racial composition of others within that system. These findings also suggest that hospital systems should prioritize gender equity programs that use an intersectionality framework, through which all women HCWs—including those who occupy different occupations, come from backgrounds underrepresented in medicine, and are caregivers—can feel supported and valued in their multiple and overlapping identities by their organization and team [42].

Our findings also suggested that gender discrimination against women HCWs may contribute to social and professional isolation, blunted professional growth, and inequitable salaries. Similarly, a study including data from the Association of American Medical Colleges on all medical graduates from 1979 to 2013 found that women physicians were less likely to be promoted to associate, full professor, or department chair, compared with men [9]. They also found that gender differences in promotion to full professor increased over time. The lack of women in leadership roles in the hospitals can have lasting impacts on the next generation of women providers; for example, women physicians-in-training have reported that the lack of women mentors in leadership roles is a significant barrier for them to advance their own careers [23]. The inequitable advancement of women HCWs has been linked to gender-based segregation of certain HCW professions and specialties in medicine [43]. Other studies have also identified persistent gender wage gaps among HCWs serving the same role [4–8]. Taken together, these findings suggest that hospital-based gender equity programs that validate and respond to experiences of gender discrimination and prioritize the career development of women HCWs, especially women HCWs of color, can address inequitable leadership opportunities and provide more mentorship opportunities for the next generation of women HCWs [42]. These programs should also implement or improve systematic approaches to identifying and remedying pay and promotion discrepancies between women and men HCWs, such as through auditing, salary transparency, and improving access to affordable childcare [44].

Our study was strengthened by its use of mixed methods to explore both the breadth and depth of gender discrimination among women HCWs [19]. By merging our quantitative and qualitative findings, we made novel interpretations for how gender discrimination impacts women HCWs. Our study was also strengthened by its timing of data collection; asking women HCWs to detail their experiences of gender discrimination during the COVID-19 pandemic highlighted the ways in which gender discrimination is particularly upsetting during a time when women HCWs are making significant sacrifices for their patients and organizations. However, our study was limited by its use of a single item to assess gender discrimination frequency; additional studies may benefit from using a measure that also characterizes the different experiences of gender discrimination based on our qualitative findings. Additionally, our study used a convenience sampling approach, which prohibited us from assessing the response rate of the survey, whether certain types of HCWs were more likely to respond than others, and if selection bias was present. Thus, studies that use random sampling may be better positioned to assess prevalence of gender discrimination among women HCWs. Furthermore, the majority of our sample was White, and additional studies that have a more diverse sample are needed to understand how gender discrimination is experienced differently for women HCWs of color. We assessed gender by using sex-based indicators, i.e., female, male, trans-female, trans-male, and non-binary, which could have introduced mismeasurement of gender into our study. Future studies should use more appropriate categories for gender, i.e., woman, man, trans-woman, trans-man, and non-binary. Lastly, this study only assessed gender discrimination for women HCWs, and additional studies are warranted that characterize experiences of gender discrimination for HCWs identifying as gender minorities and men.

## Conclusion

Our study underscores the severity of gender discrimination against women HCWs. We found that certain aspects of the pandemic exacerbated gender discrimination, such as childcare shortages, while other factors were chronic stressors, such as inequitable career advancement. Thus, our study has important implications for improving equity and inclusion during and outside of health crises. Hospital systems should prioritize gender equity programs that use an intersectionality framework [42], have measurable goals [45], facilitate the career development of women HCWs, especially women HCWs of color [42], and implement systematic approaches to identifying and remedying pay and promotion discrepancies between women and men HCWs [44].

## Supporting information

S1 File Survey questions. (DOCX)
