# Supplementary material for: Gender discrimination among women healthcare workers during the COVID-19 pandemic: Findings from a mixed methods study
Source: PLoS One. 2023 Feb 6;18(2):e0281367. doi: 10.1371/journal.pone.0281367 (PMC9901797; doi:10.1371/journal.pone.0281367)
Supplement: S1 File — (DOCX) [file pone.0281367.s001.docx]

Supplemental File. Survey questions.

What is your age?

________________________________________________________________

What is your gender?

- Male
- Female
- Transgender male
- Transgender female
- Non-binary
- Other __________________________________________________

What is your race/ ethnicity? Check all that apply.

- Hispanic or Latino
- White
- Black or African American
- Asian
- American Indian or Alaska Native
- Native Hawaiian or Pacific Islander
- Other __________________________________________________

What is your marital status?

- Single
- Married
- Divorced
- Widowed

What is your job in the hospital?

▼ Administrator ... Other

Please specify what your job is:

________________________________________________________________

| 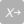 |
| --- |

Do you currently work with patients with suspected or confirmed COVID-19?

- No
- Yes, I work on COVID patient care remotely only (not face-to-face)
- Yes, I work directly with COVID patients

| 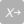 |
| --- |

Prior to the COVID-19 pandemic, were you diagnosed with any of the following mental health conditions? Check all that apply.

- Depression
- Generalized anxiety disorder
- PTSD
- Panic disorder
- Obsessive compulsive disorder
- Eating disorder
- Alcohol use disorder
- Substance use disorder
- Insomnia
- ADHD
- Other __________________________________________________
- None

State of the hospital/clinic where you work

▼ Alabama ... Wyoming

End of Block: Hospital questions

Start of Block: Social support

Since the COVID-19 pandemic, could you have used more emotional support than you received?

- Yes, a lot more
- Yes, some more
- Yes, a little more
- No

| 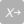 |
| --- |

Do you have children who require childcare?

- Yes
- No

| 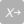 |
| --- |

Since COVID-19, could you have used more support with childcare than you received?

- No, I have enough childcare support.
- Yes, I need a little bit more childcare support.
- Yes, I need a lot more childcare support.

End of Block: Social support

Start of Block: Team cohesion

How much do you agree with the following statements:

|  | Strongly Disagree | Disagree | Neutral | Agree | Strongly Agree |
| --- | --- | --- | --- | --- | --- |
| When there is conflict in my hospital/ clinic team, the people involved usually talk it out and resolve the problem successfully. |  |  |  |  |  |
| All of the staff participates in important decisions about clinical operations. |  |  |  |  |  |
| My hospital team has been a great source of support to get through the pandemic. |  |  |  |  |  |
| The staff members of this department very frequently feel overwhelmed by the work demands. |  |  |  |  |  |
| Supervisors are available for consultation on problems. |  |  |  |  |  |
| My supervisor acknowledges the work that I do for the team. |  |  |  |  |  |
| I am inspired by the work of my team and colleagues. |  |  |  |  |  |

End of Block: Team cohesion

Start of Block: Gender discrimination

Since January 2020, how often have you been treated unfairly based on your gender?

- Never
- Once in awhile
- Sometimes
- A lot
- Most of the time
- Almost all the time

Please describe the instance(s) when you were treated unfairly based on your gender since January 2020. What happened and how did it make you feel?

________________________________________________________________

________________________________________________________________

________________________________________________________________

________________________________________________________________

________________________________________________________________

End of Block: Gender discrimination

Start of Block: General Ethnic Discrimination Scale

| 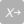 |
| --- |

We are interested in your experiences with racism. As you answer the questions below, please think about your experiences since the beginning of the pandemic, from January 2020 until now. For each question, please choose the answer that best captures the things that have happened to you.

|  | Never | Once in a while | Sometimes | A lot | Most of the time | Almost all the time | N/A |
| --- | --- | --- | --- | --- | --- | --- | --- |
| Since January 2020, how often have you been treated unfairly by **teachers and professors** because of your race/ethnic group? |  |  |  |  |  |  |  |
| Since January 2020, how often have you been treated unfairly by **your employers, bosses and supervisors** because of your race/ethnic group? |  |  |  |  |  |  |  |
| Since January 2020, how often have you been treated unfairly by **your co-workers, fellow students and colleagues** because of your race/ethnic group? |  |  |  |  |  |  |  |
| Since January 2020, how often have you been treated unfairly by **patients** because of your race/ethnic group? |  |  |  |  |  |  |  |
| Since January 2020, how often have you been treated unfairly by **people in service jobs (by store clerks, waiters, bartenders, bank tellers and others)** because of your race/ethnic group? |  |  |  |  |  |  |  |
| Since January 2020, how often have you been treated unfairly by **strangers** because of your race/ethnic group? |  |  |  |  |  |  |  |
| Since January 2020, how often have you been treated unfairly by **people in helping jobs (by doctors, nurses, psychiatrists, case workers, dentists, school counselors, therapists, social workers and others)** because of your race/ethnic group? |  |  |  |  |  |  |  |
| Since January 2020, how often have you been treated unfairly by **neighbors** because of your race/ethnic group? |  |  |  |  |  |  |  |
| Since January 2020, how often have you been treated unfairly by **institutions (schools, universities, law firms, the police, the courts, the Department of Social Services, the Unemployment Office and others)** because of your race/ethnic group? |  |  |  |  |  |  |  |
| Since January 2020, how often have you been treated unfairly by **people that you thought were your friends** because of your race/ethnic group? |  |  |  |  |  |  |  |
| Since January 2020, how often have **you been accused or suspected of doing something wrong (such as stealing, cheating, not doing your share of work, or breaking the law)** because of your race/ethnic group? |  |  |  |  |  |  |  |
| Since January 2020, how often have **people misunderstood your intentions and motives** because of your race/ethnic group? |  |  |  |  |  |  |  |
| Since January 2020, how often did **you want to tell someone off for being racist towards you but didn't say anything?** |  |  |  |  |  |  |  |
| Since January 2020, how often have **you been really angry about something racist that was done to you?** |  |  |  |  |  |  |  |
| Since January 2020, how often have **you been forced to take drastic steps (such as filing a grievance, filing a lawsuit, quitting your job, moving away, and other action) to deal with some racist thing that was done to you?** |  |  |  |  |  |  |  |
| Since January 2020, how often have **you been called a racist name**? |  |  |  |  |  |  |  |
| Since January 2020, how often have **you gotten into an argument or a fight about something racist** that was done to you or done to another member of your race/ethnic group? |  |  |  |  |  |  |  |
| Since January 2020, how often have **you been made fun of, picked on, pushed, shoved, hit, or threatened with harms** because of your race/ethnic group? |  |  |  |  |  |  |  |

| Page Break |  |
| --- | --- |

| 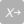 |
| --- |

How different would your life be now if you had not been treated in a racist and unfair way?

- The same as it is now
- A little different
- Different in a few ways
- Different in a lot of ways
- Different in most ways
- Totally different
- N/A

End of Block: General Ethnic Discrimination Scale
